# Potential Transferability of Economic Evaluations of Programs Encouraging Physical Activity in Children and Adolescents across Different Countries—A Systematic Review of the Literature

**DOI:** 10.3390/ijerph111010606

**Published:** 2014-10-15

**Authors:** Katharina Korber

**Affiliations:** 1 Munich School of Management and Munich Center of Health Sciences, Ludwig-Maximilians-University, Munich 80539, Germany; E-Mail: korber@bwl.lmu.de or Katharina.korber@helmholtz-muenchen.de; 2 Institute for Health Economics and Health Care Management, Helmholtz Zentrum München (GmbH)—German Research Center for Environmental Health, Neuherberg 85764, Germany

**Keywords:** review, economic evaluation, physical activity, children and adolescents, primary prevention, health promotion, transferability

## Abstract

Physical inactivity is an increasing problem. Owing to limited financial resources, one method of getting information on the cost-effectiveness of different types of prevention programs is to examine existing programs and their results. The aim of this paper is to give an overview of the transferability of cost-effectiveness results of physical activity programs for children and adolescents to other contexts. Based on a systematic review of the literature, the transferability of the studies found was assessed using a sub-checklist of the European Network of Health Economic Evaluation Databases (EURONHEED). Thirteen studies of different physical activity interventions were found and analyzed. The results for transferability ranged from “low” to “very high”. A number of different factors influence a program’s cost-effectiveness (*i.e*., discount rate, time horizon, *etc.*). Therefore, transparency with regard to these factors is one fundamental element in the transferability of the results. A major point of criticism is that transferability is often limited because of lack of transparency. This paper is the first to provide both an overview and an assessment of transferability of economic evaluations of existing programs encouraging physical activity in children and adolescents. This allows decision makers to gain an impression on whether the findings are transferable to their decision contexts, which may lead to time and cost savings.

## 1. Introduction

Over the last few decades, children, adolescents, and adults have all become less physically active. Reasons for physical inactivity can be work related for example (especially for adults). Another possible reason for the increase in sedentary behavior is the availability of a broad range of media products and their usage, especially by children and adolescents [1,2]. In their study of screen-related behaviors of grade 5 and 6 students, He *et al.* found that boys had a total average screen time of 3.6 h per day while girls spent an average of 3.1 h per day on screen-related activities [2].

A physically inactive lifestyle is an important behavioral risk factor, which is accompanied by a number of health risks. For example, it is related to an increased risk of cardiovascular disease (CVD) [3,4], one of the leading causes of death in developed countries [5]. Additionally, in developed countries with high energy intakes, the lack of physical activity can contribute to the development of overweight or even obesity as a consequence of higher energy intake than energy expenditure even in infancy. Overweight and obesity are themselves risk factors for a range of chronic diseases. They can also intensify other chronic conditions, such as hypertension and high cholesterol. Additionally, adult weight gain is related to an increase in the risk of coronary heart disease and type 2 diabetes mellitus [6,7].

Owing to limited financial resources, only effective and, where possible, only cost-effective intervention measures should be adopted in health promotion and primary prevention [8], as maximizing the possible effects and benefits with the given financial resources is a desirable economic and health economic goal. As it is complex and not always possible to estimate the costs of physical activity programs in advance, one way of getting information on the cost-effectiveness of different approaches can be to examine existing program evaluations. There are a number of different factors that influence the cost-effectiveness of a program, such as the underlying health care system, epidemiological patterns, or simply prices [9]. To assess the transferability of results from other countries to one’s own context, close attention has to be paid to a number of different criteria.

When looking at the results of already existing studies to support decision makers or researchers on whether or not to transfer the results to the own context, it is necessary to know as many details as possible of those studies and the factors influencing cost-effectiveness and transferability. There is still limited evidence on the assessment of transferability in the research field of primary prevention and health promotion focusing on physical activity in children and adolescents. Based on this, the aim of this paper is to give an overview of the transferability of cost-effectiveness results of programs encouraging physical activity in children and adolescents across different countries.

## 2. Methods

### 2.1. Search Process

To identify the health economic evaluations needed to answer the review question, a comprehensive systematic review of the literature was conducted including all relevant literature published before 30 June 2014. The following databases were used to find relevant literature: PubMed, Web of Science, Centre for Reviews and Dissemination (CRD) databases (DARE, NHS EED, HTA), and EconLit.

For the database PubMed, the following search terms were used: (((economic evaluation) OR (cost-effectiveness) OR (cost-benefit) OR (cost-utility) OR (economics)) AND (physical activity OR sport OR movement OR sedentary behavior) AND (Humans[MeSH] AND (English[lang] OR French[lang] OR German[lang]) AND (infant[MeSH] OR child[MeSH] OR adolescent[MeSH]))). This query was used analogously in the other databases, adapted to the given possibilities of the particular database. Additionally, citation tracking in Google Scholar was used as well as a manual search of the reference lists of included studies.

### 2.2. Inclusion/Exclusion Criteria

Secondary prevention measures in which the target group is already overweight or obese were excluded; there are already other reviews that concern themselves with this topic [10]. However, primary prevention measures for obesity that involve all infants regardless of initial weight were considered. Studies were excluded when physical activity was used as a secondary prevention measure for already existing diseases in the target group. The criterion “infants and adolescents” was always essential for the search. Accordingly, only those studies that fulfilled this criterion were taken into account. Studies that examine measures for parents, in which their offspring only benefit indirectly, have therefore not been included in the report. Additionally, only English, German, and French publications were included. Publications on developing countries were omitted, because problems with physical inactivity are currently uncommon in such countries. Studies that only analyze effects, as well as reviews and meta-analyses, such as those of Wu *et al.* [11], were also excluded. Only original studies were included in this review.

### 2.3. Data Extraction

To summarize the fundamental methodological elements of the health economic evaluations included in the review, data extraction was performed using detailed information about major characteristics of the particular studies (author, year, country, intervention components, study objective, target group, setting, study design, perspective, time horizon, discounting, the effects measured, and details of the costs (price year, currency, cost categories)).

### 2.4. Assessment of Transferability

As can be found in Goeree *et al.* [12], there are a variety of different checklists to assess the transferability of health economic evaluations, such as the decision chart by Welte *et al.* [13], or different checklists for transferability, such as those from Boulenger *et al.* [14], Drummond *et al.* [15], and Urdahl *et al.* [16].

The assessment in this review is based on a sub-checklist of the European Network of Health Economic Evaluation Databases (EURONHEED). This checklist was developed with the objective of providing decision makers (especially in Europe) with information on the reliability and relevance of economic evaluations and helping to identify those that are potentially transferable by giving an indication of the level of transferability [14].

It was adapted to modeling studies, finally consisting of 18 questions considering the following aspects: health technology, setting, perspective, study population, modeling, effectiveness, benefit measure, costs, and discussion. The possible scoring of the answers was 1 for “yes”, 0.5 for “partially”, and 0 for “no/no information”. Additionally, there was the option N/A for not applicable. Questions with the answer “N/A” were excluded from the overall transferability scoring.

The summary score was then calculated using the following formula: [1/(*n* – *x*)]Σ*_i_S_i_* × 100 [14], rounded to the nearest 1%; *i* = 1, .., *n* , *n* is the number of questions, *x* is the number of questions for which the response was N/A, and *S* is the score for each question. The ranking for transferability was based on the following ranges of the achieved scores:

- Low: <50%;
- Medium: 50 ≤ 60%;
- High: 60%–80%;
- Very high: >80%.

## 3. Results and Discussion

### 3.1. Results

#### 3.1.1. Results of the Research

The research includes all studies that were found in the databases and with additional research (citation tracking, manual search), as described in the methods section before 30 June 2014. In total, 1827 hits were retrieved. Some 1814 articles were excluded after screening the abstract and title or the full text, as they were duplicates or did not fulfill the relevant inclusion criteria. An overview of the search process is given in Table 1.

**Table 1:** Electronic databases searched for economic evaluations.

Database	Hits	Excluded Articles ^a^	Remaining
PubMed	1504	1497	7
Web of Science	225	222	3
CRD databases (DARE, NHS, EED, HTA)	37	34	3
EconLit	61	61	0
All databases	1827	1814	13

^a^ Covering only effects, concerning only developing countries, referring only to secondary prevention; exclusion of duplicates.

In total, 13 studies were found that fulfilled the inclusion criteria. Twelve different interventions aiming to increase physical activity in children and adolescents (and to some extent better nutrition and a healthier lifestyle in general) were analyzed focusing on the prevention of overweight and obesity [17,18,19,20,21,22,23,24,25,26,27,28]. One of the interventions was also examined with regard to the prevention of disordered weight control behaviors (DWCB) [29].

#### 3.1.2. Study Characteristics and Key Findings

Table 2 lists all the economic evaluations described above, including the condensed key information of: author, year of publication, country, type, aim and duration of intervention, target group, study design, perspective, time horizon, discounting rate, measured effects, and cost components.

As shown in Table 2, 13 studies focusing on the economic aspects of 12 different programs, aimed at encouraging physical activity in children and adolescents, were found and analyzed. Five publications focused on programs in the USA, four on programs in Australia, two on programs in Germany, and the final two on programs in the United Kingdom and New Zealand.

As Table 2 shows, the studies analyzed different types of intervention, but all interventions had the common goal of encouraging physical activity in children and/or adolescents. The duration of the programs was also different. At only 6 weeks, the shortest intervention was the media campaign, analyzed by Peterson *et al.* [25]. The other interventions that were analyzed all had a duration of one or two years, and some of them are still ongoing (without further documentation of the health economic aspects) (e.g., [21]).

Eight of the publications cited the primary prevention of overweight as being the major aim of the intervention [17,18,19,20,21,23,24,27,28], two publications focused on exercise promotion in general [25,26], and the last publication reported the primary prevention of DWCB as the major aim of the intervention [29]. It is interesting to note that this last study [29] was based on the same program (Planet Health) as the study of Wang *et al.*, 2003 [28], with one of them examining the prevention of overweight and the other the prevention of DWCB. This shows that programs encouraging physical activity can also have other effects besides preventing obesity and that this can have an influence on the economic impact of such programs.

Only interventions for children and adolescents were considered in this review; therefore, children and adolescents aged between 4 and 17 years participated in the prevention programs.

Most of the interventions took place in a school setting (seven interventions) [17,21,23,24,27,28,29], but one of them was in a community setting [26], and four had a combination of a school and a community setting [18,19,20,22], whereas the final intervention was designed for society [25].

When looking at the study design, five studies analyzed the economic aspects of the intervention programs [17,22,23,25,27] with cost-effectiveness analyses [17,22,23,25,27], and eight studies used a model approach [18,19,20,21,24,26,28,29]. Of the studies that have a model approach, five performed cost-utility analyses [18,19,20,21,26], and three performed cost-effectiveness analyses and additionally reported the net benefit of the intervention [24,28,29].

Most of the studies calculated costs and effects from a societal perspective. Only the studies of Peterson *et al.*, and Pringle *et al.* [25,26] did not report a perspective but only the type of costs that were considered. These two studies reported neither the time horizon for costs and effects nor the discounting rate. Four studies conducted by Moodie *et al.* [18,19,20,21] calculated a lifetime model and discounted costs and benefits at 3% per year. In the study by Brown *et al.* [24] and that by Wang *et al.*, 2003 [28], the same rate for discounting costs and effects was used as in the four above-mentioned studies, but the time horizon for the model was 25 years [24,28]. The second modeling study by Wang *et al.*, (2011) [29] also used 3% for discounting, but a time horizon of only 10 years. The four remaining studies are intervention studies, two of them with a time horizon of 1 year and therefore without discounting costs and effects [23,27], one with a time horizon of 4 years and a discount rate of 5% for the costs [22], and one reported details of neither the time horizon nor the discounting rate [17].

The reported effect categories ranged from (clinical) parameters such as changes in body mass index (BMI) [17,18,19,20,21,23], reduction in body fat [27], waist-to-height ratio [23], waist circumference [23], prevented cases of overweight [22,24,28] or DWCB [29], changes in physical activity and energy expenditure [17,19,20,21,25,26] to health-related quality of life (QALY, DALY) [18,20,22,24,26,28,29] and even monetarily assessed net benefit [24,28,29].

Looking at the costs, they were usually given in the respective national currency with price years ranging from 1996 to 2010.

#### 3.1.3. Transferability Assessment

Table 3 gives an overview of the transferability assessment for the 13 relevant studies described above. The studies are sorted by publication year. For the five studies using intervention results [17,22,23,25,27] and no modeling, the scoring was made without the two questions concerning the modeling (M1, M2); for the other studies, these questions were included in the score. There were four questions in which all studies scored 1 (HT1, SE2, E5, C9) and one question where none of the studies scored at all (S1). In all the other questions, the scores varied from 0 to 0.5 or from 0 to 1.

Six of the studies reached a total score of more than 80%, which indicates a very high level of transferability to other contexts with the given information [18,20,23,24,27,28]. Four of the studies reached a score of between 60% and 80%, which can be interpreted as high transferability based on the underlying information [19,21,22,29]. The remaining three studies scored in the range of 44% to 60% [17,25,26].

### 3.2. Discussion

#### 3.2.1. Major Findings

Transferability assessment of already existing economic evaluations can be a cost- and time-saving option to gain an impression of the effectiveness and cost-effectiveness of a corresponding or similar program in one’s own context. Sometimes, when performing an economic evaluation of local studies is not feasible, it might even be the only possibility [14].

There are a number of different factors influencing the results regarding the cost-effectiveness [9] of a primary prevention program that are also fundamental for the transferability of the results. Therefore, the most important factors for transferability regarding prevention programs in the field of physical activity for children and adolescents are discussed here.

**Table 2:** Study description and overview of economic evaluations of physical activity programs (sorted by publication year).

Author/Year/(Country)	Intervention Components	Aim	Target/Age Group	Setting	Study Design	Perspective, Time Horizon, Discounting	Measure of Effects	Price Year/Currency Unit, Considered Cost Categories	Result
Wang *et al.*, 2003 (USA) [28]	Interdisciplinary approach, lessons, sport materials, wellness, teacher training	Prevention of overweight	Children 6th–8th school year, 11–13 years	School	CEA, using a model approach (calculating additional benefit)	Society, modeling over a 25 year period, costs and benefits both at 3%	Cases of adult overweight prevented (5.805), QALYs (4.13)	1996, USD, intervention costs, avoided treatment costs, avoided productivity loss costs	USD 4305/QALY saved
Brown *et al.*, 2007 (USA) [24]	Physical activity, nutrition	Prevention of overweight	Children, grades three, four and five, 8–11 years	School	CEA, using a model approach (calculating additional benefit)	Society, modeling over a 25 year period, costs and benefits at 3%	Cases of adult overweight prevented (40–64 years), QALYs saved	2004, USD, intervention costs, avoided treatment costs, avoided productivity loss costs	USD 900/QALY saved
Wang *et al.*, 2008 (USA) [27]	After-school program: physical activity, healthy snacks, support with homework, and “academic enrichment”	Prevention of overweight	Children, elementary school, 6–10 years	School	CEA, using intervention results	Society, 1 year, not stated	% Reduction in body fat	2003, USD, intervention costs, after-school care costs without intervention	USD 417 per % point body fat reduction
Peterson *et al.*, 2008 (USA) [25]	Media campaign	Exercise promotion	Teenagers, 12–17 years	Society	CEA, using intervention results	Only program costs, not reported, not stated	Questionnaire, extrapolated to population: “contemplated doing more exercise”, “has done more exercise”	No price year, USD, development costs of the program and costs for “product placement”	Cost per person who did more exercise: between USD 5.11 and USD 153.19 for the individual sections of the campaign, USD 8.87 for the whole campaign
Moodie *et al.*, 2009 (AUS) [19]	“Walking School Bus” encouraging physical activity	Prevention of overweight	Children, 5–7 years	School/Community	CUA, using a model approach	Society, lifetime, costs and benefits both at 3%	Reduction in BMI, increase in physical activity, energy expenditure	2001, AUD, total costs	Lifetime DALYs, Cost per: - DALY saved: AUD 760,000 (net; gross: AUD 770,000) - BMI unit saved: AUD 87,000
McAuley *et al.*, 2010 (NZ) [22]	Nutrition and physical activity	Prevention of overweight	Children, 5–12 years	School/Community	CEA, using intervention results	Society, 4 years, costs at 5%	Weight gain avoided, QALY	2006, NZD, no development costs, total costs	NZD 664–1708 per kg avoided weight gain
Pringle *et al.*, 2010 (UK) [26]	Activity classes, free swimming activities	Exercise promotion	Population (children 10–17 years)	Community	CUA, using a model approach	Key implementation and running costs, not stated, not stated	Change in MPA, QALY	2003, GBP, costs/completer improving MPA	GBP 94–103/QALY gained
Moodie *et al.*, 2010 (AUS) [21]	After-school care for children from 3 to 5 pm including a physical activity program	Prevention of overweight	Children, primary school, 5–11 years	School	CUA, using a model approach	Society, lifetime, costs and benefits both at 3%	Reduction in BMI, increase in physical activity, energy expenditure	2001, AUD, total cost	Lifetime DALYs, Gross cost per: - DALY saved: AUD 82,000 (net; gross: AUD 90,000)/ - BMI unit saved: AUD 8200
Kesztyüs *et al.*, 2011 (GER) [23]	Health education, physical activity breaks, and parent involvement	Prevention of overweight	Children, primary school, second grade, 7–8 years	School	CEA, using intervention results	Society, 1 year,/not stated	Differences in waist-to-height ratio, waist circumference, and BMI	2008, EUR, total intervention costs, intervention costs per child	ICER (WC) = EUR 11.11 per cm prevented; ICER (WHtR) = EUR 18.55 per unit prevented
Wang *et al.*, 2011 (USA) [29]	Interdisciplinary approach, lessons, sport materials, wellness, teacher training	Prevention of DWCB	Children (6th–8th school year), 11–13 years	School	CEA, using a model approach (calculating additional benefit)	Society, 10 years, costs and benefits both at 3%	DWCB avoided, QALYs	2010, USD, total costs	USD 2966/QALY saved
Moodie *et al.*, 2011 (AUS) [20]	Lessons, information evenings, promotion of the program	Prevention of overweight	Children, 5th and 6th school years, 10–11 years	School// Community	CUA, using a model approach	Society, lifetime, costs and benefits both at 3%	Reduction in BMI, increase in physical activity, energy expenditure, DALY	2001, AUD, total costs	Lifetime DALYs, Cost per: - DALY saved: AUD 117,000 (net; gross: AUD 125,000) - BMI unit saved: AUD 13,000
Moodie *et al.*, 2013 (AUS) [18]	Interdisciplinary approach, including nutrition and physical activity and reducing screen time	Prevention of overweight	Children, 4–12 years	School//Community	CUA, using a model approach	Society, lifetime, costs and benefits both at 3%	Reduction in BMI, DALY	2006, AUD, total costs	Lifetime DALYs, Cost per: - DALY saved: AUD 20,227 (net; gross: AUD 22,978) - BMI unit saved: AUD 399
Krauth *et al.*, 2013 (GER) [17]	3 additional PE lessons per week	Prevention of overweight	Children, primary school	School	CEA, using intervention results	Society, not stated, not stated	Reduction in BMI, increase in physical activity	No price year, EUR, intervention costs, intervention costs per child per school year	EUR 619/student per year for significant results

Abbreviations: AUD: Australian dollar; AUS: Australia; BMI: body mass index; CBA: cost-benefit analysis; CE: cost-effective; CEA: cost-effectiveness analysis; CUA: cost-utility analysis; DALY: disability-adjusted life year; DWCB: disordered weight control behaviors; EUR: Euro; GBP: UK pound; MPA: moderate physical activity; NHS: National Health Service; NZ: New Zealand; NZD: New Zealand dollar; PE: physical education; QALY: quality-adjusted life year; USA: United States of America; USD: US dollar; WC: waist circumference; WHtR: waist-to-height ratio; WTP: willingness-to-pay.

**Table 3:** Assessment of potential transferability of economic evaluations—overview.

1 = Yes, 0.5 = Partly, 0 = No/No Information, N/A = Not Applicable		Wang *et al.*, (2003)	Brown *et al.*, (2007)	Wang *et al.*, (2008)	Peterson *et al.*, (2008)	Moodie *et al.*, (WSB-2009)	McAuley *et al.*, (2010)	Pringle *et al.*, (2010)	Moodie *et al.*, (AAS-2010)	Kesztyüs *et al.*, (2011)	Wang *et al.*, (2011)	Moodie *et al.*, (TS-2011)	Moodie *et al.*, (BAEW-2013)	Krauth *et al.*, (2013)
Health technology	HT1. Is the intervention described in sufficient detail?	1	1	1	1	1	1	1	1	1	1	1	1	1
	HT2. Is (are) the comparator(s) described in sufficient detail?	0.5	0.5	1	0	0.5	0.5	0.5	0.5	0.5	0.5	1	1	0.5
Setting	SE2. Is (are) the country(ies) in which the economic study took place clearly specified?	1	1	1	1	1	1	1	1	1	1	1	1	1
Perspective	P1. Did the authors correctly state which perspective they adopted for the economic analysis?	1	1	1	0	1	1	0	1	1	1	1	1	1
Study population	SP1. Is the target population of the health technology clearly stated by the authors or when it is not done can it be inferred by reading the article?	1	1	1	0.5	1	1	0.5	1	1	1	1	1	0.5
	SP3. Does the article provide sufficient detail about the study sample(s)?	0.5	0.5	0.5	0.5	0.5	0.5	0.5	0.5	0.5	0.5	0.5	0.5	0
Modeling	M1. If a model is used is it described in detail?	1	1	N/A	N/A	1	N/A	0.5	1	N/A	0.5	0.5	0.5	N/A
	M2. Are the origins of the parameters used in the model given?	1	1	N/A	N/A	1	N/A	0.5	1	N/A	1	1	1	N/A
Effectiveness	E5. Have the principal estimates of effectiveness measures been reported?	1	1	1	1	1	1	1	1	1	1	1	1	1
	E7. Does the article provide the results of a statistical analysis of the effectiveness results?	0.5	0.5	0.5	0.5	0.5	0.5	0.5	0.5	1	0.5	0.5	0.5	0.5
Benefit measure	B5. Is the level of reporting of benefit data adequate (incremental analysis, statistical analyses)?	1	1	0.5	0.5	0.5	0.5	0.5	1	1	0.5	1	1	0.5
Costs	C1. Are the cost components/items used in the economic analysis presented?	1	1	1	0.5	1	1	0	1	1	1	1	1	1
	C5. Are unit prices for resources given?	1	0.5	1	0	1	1	0	0.5	1	1	1	1	0
	C6. Are costs and quantities reported separately?	1	1	1	0	1	1	0	0.5	1	1	0.5	0.5	1
	C7. Is the price year given?	1	1	1	0	1	1	1	1	1	1	1	1	0
	C9. Is the currency unit reported?	1	1	1	1	1	1	1	1	1	1	1	1	1
Discussion	S1. Are quantitative and/or descriptive analysis conducted to explore variability from place to place?	0	0	0	0	0	0	0	0	0	0	0	0	0
	O1. Did the authors discuss caveats regarding the generalizability of their results?	1	0.5	0.5	0.5	0.5	0.5	0.5	0.5	1	0.5	0.5	0.5	0.5
Score (%) *		86	81	81	44	78	78	50	78	88	78	81	81	59
Transferability of the study to other contexts		Very high	Very high	Very high	Low	High	High	Medium	High	Very high	High	Very high	Very high	Medium

* The summary score was calculated using the following formula: [1/(n–*x*)] Σ*_i_* *S_i_* × 100, [14], rounded to the nearest 1%; *i* = 1,.., *n*, *n* is the number of questions, *x* is the number of questions for which the response was N/A, and *S* is the score for each question. <50% low, 50% ≤ 60% medium, 60%–80% high, >80% very high.

First, the interventions can differ greatly, making a sufficient description of the intervention necessary. All studies found in this review described the intervention they examined in sufficient detail, but only three described the comparators sufficiently [18,20,27]. Another important factor is the setting in which the program is implemented. In this review, this was a school setting [17,21,23,24,27,28,29], a community setting [26], a combination of school and community [18,19,20,22], or society [25], and this was clearly stated by all the authors. Knowing the country in which the study took place is essential, as the underlying health care system and country-specific prices become relevant when calculating avoided treatment costs. The implementation costs of programs also depend on local wages and prices. Particularly for cost calculations, it is important that there is information on quantities (two studies did not score [25,26], four studies scored partially [18,19,20,21], the others scored fully), unit prices of resources (three studies did not score in this category [17,25,26], three scored partially [19,21,24] and the others scored fully), price year (two studies did not score at all [17,25]), and currency (all scored “1”).

Effectiveness data can also be country specific [9] and need to be given in adequate detail so that the results can be made transferable to other countries. In the checklist, this is represented by questions E5 and E7 and was included in all studies, in detail for E5 (all scored fully), and at least partially for E7 (all scored “0.5”, except one that scored “1” [23]).

In none of the studies considered in this review was there an analysis to explore variability from place to place. Caveats regarding the generalizability of results were clearly discussed by the authors in two studies [23,28] and only implicitly in the other studies.

In total, this leads to a wide variation in the transferability of the study results ranging from “low” to “very high” with everything in between.

One major point of criticism of this result is the fact that some studies might only have a low score in the assessment because of lacking transparency but not because of lacking study quality. This can for example result from the studies being published in a variety of different journals with journal-specific standards for publication (e.g., space limitations). The study by Krauth *et al.* [17] is an example of this problem. It can be classified as a health economic evaluation itself (and then reach a relatively low score), but in fact it can also be seen as a sort of study protocol for a subsequent modeling study that has not been published yet and might be more detailed. This, and the fact that it is relatively short, might be the reason for the relatively low score in this transferability assessment.

#### 3.2.2. Limitations of This Review

The main limitations of this review are that the collection of publications was limited to those referenced in the databases PubMed, Web of Science, CRD databases (DARE, NHS EED, HTA), and EconLit. Citation tracking in Google Scholar and an additional manual search were used to broaden the search. A further restriction was made by only including publications in English, German, and French and excluding publications in other languages. The last update for searching the databases was 30 June 2014, so later publications are not captured by this review.

Using the sub-checklist of the European Network of Health Economic Evaluation Databases (EURONHEED) [14] for the assessment of transferability may be another limiting factor in this review. It is only a short version of the original assessment list, and so other items that are part of the original list have not been considered here. Based on the publication by Boulenger *et al.*, however, the items used in the sub-checklist were the most important in assessing transferability [14]. Another point of possible criticism is the fact that each item in the EURONHEED checklist is incorporated in the overall transferability score with the same weight [12,14].

Another limitation of this review is that it was conducted by only one researcher, although it would have been more objective [30] to have had two independent reviewers undertaking the extraction and appraisal of the studies.

#### 3.2.3. Comparison with Other Reviews

There are already numerous reviews focusing on the effectiveness of physical activity programs for adults as well as for children and adolescents [31,32,33], as well as several reviews of economic evaluations focusing on physical activity as a (disease-specific) secondary prevention method for both children/adolescents (for example for obesity: [34]) and adults (for example reduction of risk factors for metabolic syndrome: [35]). However, there is still a gap in the field of health economic evaluations focusing on physical activity for children. When looking at the transferability of the economic results for physical activity programs, there is only one review for elderly adults [8]. Until now, however, there has been no review concerning the transferability of health economic evaluations of programs encouraging physical activity with the target group of children and adolescents. Because economic evaluations in this research field are still limited and just starting to become more important (see study characteristics for the publication years), the assessment of the transferability of existing results to other contexts is an important research contribution. Therefore, this review was conducted to fill this research gap.

## 4. Conclusions

The first aim of primary prevention and health promotion measures should always be their effectiveness. However, faced with scarce resources, programs should also be cost-effective.

Despite an intensive review of the literature, only a few economic evaluation studies of physical activity programs in children and adolescents were found. Looking at the publication years, it can be seen that the majority of studies found in this research derive from the year 2007 onwards (only one study was published earlier, in 2003 [28]), giving the impression that this is an ongoing topic of public health research. It becomes apparent that a demand for economic evaluations of primary prevention interventions [36] is still not common in current practice, but has become more important in the last few years.

Regarding the cost-effectiveness, this overview shows that some primary prevention programs encourage physical activity at relatively low cost per QALY, which points towards the cost-effectiveness of these programs. For further programs, the relation of costs per QALY is much higher and it depends on the decision maker’s willingness to pay for a QALY whether or not these programs are cost-effective. The same applies to the programs that do not use QALYs as a measure but other health outcomes. Here again, the costs seem to be relatively low in the examined programs, but still it depends on the willingness to pay for the achieved changes in health measures, such as BMI unit or % body fat reduction.

Looking at the transferability, none of the studies scored 100%. At least 10 out of 13 studies already scored “very high” or “high” [18,19,20,21,22,23,24,27,28,29], which means that their results should be of high transferability to similar programs in other (but similar) contexts, but there are still three studies with a low or medium score for transferability [17,25,26]. As there are only a few economic evaluations of physical activity programs in general, it would be desirable to have more studies reaching a high or very high score for their transferability, so that researchers from different countries and contexts can use the results for planning possible health promotion or primary prevention programs for this target group.

During the research, it was found that an important step towards more (economic) transparency would be a more transparent documentation of the costs of development, implementation, and continuation of an intervention as well as a more transparent documentation of the effects achieved. This would lead to higher quality studies and, in some instances, also to better transferability to other contexts so that other countries can benefit from the positive or negative experiences others have made with different physical activity programs. Not needing to conduct one’s own economic evaluation would also save money to be invested instead in conducting proven effective and transferable primary prevention programs.
